# Round the Hospitals

**Published:** 1919-06-07

**Authors:** 


### ROUND THE HOSPITALS.

A large number of members of the nursing services have received recognition in the list of the King's Birthday Honours of their valuable services with the armies in France and Flanders. Those who have been awarded the Bar to the Royal Red Cross are Miss M. Alexander, Miss G. M. Allen, Miss A. I. Baird, Miss M. C. Laing, Miss L. E. M'Kay, Miss E. J. Minns, Miss M. L. Raunie, Miss U. E. Russell-Lee, Miss E. C. Schofield, Miss A. L. Walker, Miss E. M. Wilson,

**ROUND THE HOSPITALS—(*continued*).**

Miss E. G Brooke. The awards of the Royal Red Cross, First Class, number fifty-two British, seven Canadian, four Australian, one New Zealand nursing sisters and matron. The awards of the Second Class of the Order include nearly 200 British, forty Canadian, ten Australian, one New Zealand, three South African nursing sisters and matrons, among whom are a few V.A.D. nurses.

At the Investiture held on May 29 in the Quadrangle of Buckingham Palace, the King personally conferred the Decoration of the Royal Red Cross Second Class, on Sister Florence McClelland, Q.A.I.M.N.S.; on Assistant Matron Elizabeth Pettigrew, Sister Lucy Calvert, Sister Jemima Grant, Sister Agnes O'Brien, Sister Elizabeth Simson, Sister May Wilson, and Staff Nurse Winifred Jones, Q.A.I.M.N.S.R.; on Sister Elizabeth Ramage, C.N.S.; on Sister Elizabeth Best and Sister Dolors Massey, Canadian A.N.S.; and on Lady MacMillan and Miss Margaret Vaggis, V.A.D. All these ladies were subsequently received at Marlborough House by Queen Alexandra.

At the Investiture held in the Quadrangle of Buckingham Palace on May 31 the King personally conferred decorations on members of the nursing Services as follows: Bar to the Royal Red Cross, Miss Mary E. Davies, Matron Q.A.I.M.N.S.R.; Miss Annie Gill, Principal Matron T.F.N.S. Royal Red Cross, First Class, Sister Grace King and Sister Margaret Lyons, Q.A.I.M.N.S.R.; Sister Mildred Wheeler, T.F.N.S.; Matron Mabel Astley-Campbell, B.R.C.S., and Matron Marion Parsons, United States A.N.S. Royal Red Cross, Second Class.—Sister Margaret Caird, Sister Agnes Coomby, Sister Alexina Donald, Sister Lizzie Fletcher, Staff Nurse Hilda Franklin and Staff Nurse Maud Plant, Q.A.I.M.N.S.R.; Matron Antoinette Saunders, Civil N.S.; Matron Edith Johncock and Sister Ethel Dowling, B.R.C.S.; Matron Rose Phillips and Matron Maud Ruffle, St. John A.B.; Sister Silla Carr-Harris, Canadian A.N.S.; Matron Minnie Joiner, Nurse Annie Kydd and Nurse Ethel Warrington, V.A.D.

Universal interest will be stirred by the issue of the momentous Report of the Salaries Committee appointed by the Council of the College of Nursing to investigate the salaries and conditions of employment of nurses. The Committee consisted of Mrs. E. M. Field (Chairman), the Dowager Countess Brassey, Miss Rosa Barrett, Mrs. Edred Corner, R.R.C., Mrs. W. L. Courtenay, O.B.E., Miss A. C. Gibson, Miss Haldane, C.H., L.L.O., Mrs. David Macdonald, Dame Mary Whittey, G.B.E., Mr. Louis H. Dick. They held six meetings, and the sub-committees (*a*) on Conditions of Employment (*b*), on Salaries, held five additional meetings. They despatched two questionnaires, one of which was sent to 275 general hospitals, 137 Poor-Law institutions, 121 special hospitals, 569 hospitals containing less than forty beds, 139 provincial fever hospitals, twenty-nine hospitals for incurables, eight epileptic colonies, thirteen dispensaries, and six homes for defectives. The other form of inquiry was sent to 569 nursing institutions in London and the Provinces, convalescent homes and sanatoria, private nursing homes, medical officers of health and works and collieries. They received 514 replies to the first and 240 to the second form.

The manner in which this mass of information has been dealt with is worthy of all praise, and testifies to an unusual spirit of energy in the Committee, as well as to their fitness for the duties they had to perform. The brief report is a mine of information. The highest number of hours of duty shown on the returns is seventy-one hours a week on day duty and eighty-four on night duty. The lowest is fifty-two and a-half hours a week day duty and fifty-nine and a-half hours night duty. The Committee place it on record that they recommend that an average week of forty-eight hours of duty be fixed as a maximum for all nurses, such average to be taken over a period of not more than three months. Three admirably worked out illustrations of the manner in which this may be arranged are subjoined. A carefully thought out scale of minimum salaries suggested in various branches of nursing and various kinds of institutions is appended. The well-prepared tables which show the variations in salary paid for the same services in different classes of institutions are among the most remarkable statistics it has ever been our lot to peruse. The Poor-Law infirmaries come very badly indeed out of this comparison, and the London infirmaries in some parts show up worse than those in the Provinces, in Scotland, and in Ireland. Few people could have conjectured that the average salary given to matrons in London Poor-Law infirmaries containing over 500 beds is £126, while in those containing between 200 and 300 beds it is £65 10s. It is sufficient to note that the minimum salary suggested for the matron in these institutions is £350 (over 500 beds) and £250 (between 200 and 300 beds).

Thirty applications have been received from V.A.D.s who wish to take training in medicine under the scholarship scheme of the British Red Cross Society and the Order of St. John. Ten of these have passed the Selection Board and received grants. Their names are: Beatrice Turner, London (detachment 22); Florine Irwin, Dublin (30); Joan Lush, Cambridge (28); Winifred Kemp, Cape Town; Minnie Gosden, Worcester (54); Kathleen Hornby, West Lancs (38); Margaret Price Surrey (102); Laura Sanders, London (40); Barbara Finch, Dorset (36); Margaret Ritchie, London (38).

The College of Nursing is losing its excellent Registrar, Miss Ashdown, who has gained golden opinions during her comparatively short term of

**ROUND THE HOSPITALS—(*continued*).**

office. Miss Ashdown found time to edit a good manual called the "Complete System of Nursing," and has proved herself to be a very successful examiner in nursing at several London training-schools for nurses. She leaves the College to join with her sister in taking a nursing home in Devonshire Street. The vacant appointment of Registrar to the College of Nursing is one of the plums of the profession. The post of assistant-secretary, vacated last year by Miss Cowlin when she became organising secretary, is also advertised.

The number of Sister-Tutor Studentships offered by the College of Nursing has this year been raised to five. They are of the value of 100 guineas each, and afford one year's course of study to nurses who are already certificated. Four are tenable at the Household and Social Science Department, King's College for Women, University of London. Full particulars will be given on application to the Secretary of the College of Nursing, 7 Henrietta Street, Cavendish Square, W. 1. The examination last year was in August, and twelve students entered. It is probable that a much larger number will compete this year, since so many sisters after war work are seeking fresh openings. It is noticeable in the Annual Report of the College that the Selection Committee is stated to have paid special attention in awarding the scholarships to the past experience and references of the candidates, the total number of marks gained in the qualifying examination being only one factor in the selection of the successful competitor. This should reassure the many aspirants who are "examination shy."

Good news was announced at the recent meeting of the Metropolitan Asylums Board. It was stated that the Conciliation Board, representing asylum authorities and employees, had agreed upon a general recommendation in favour of a working week not exceeding sixty hours inclusive of meal-times for all indoor staffs. The governing authorities of the different institutions, in consultation with their staffs, are to carry the suggestion into effect in the manner which best suits them. The Committee report also that a Conference is being arranged in which representatives of the M.A.B. will take part. The question of wages has yet to be considered. The concession of a sixty-hours' week inclusive of meals to asylum workers cannot therefore now be long delayed. It will go far to make their lives worth living.

The Report of the Asylum Workers' Association for 1918 is a pathetic document, for it is probably the last. Although the membership has slightly risen it barely exceeds 2,000 and the trifling subscription of 1s. 6d. by no means suffices to keep going "The Asylum News," which is distributed free to all members, and to defray the small expenses of management. Amalgamation with the Medico-Psychological Association has been suggested, and if that is effected, the Association will become an Asylum Workers Section of that body. The Gold Medal of the Society was presented to Mr. J. H. Hodges, Mrs. S. A. Hulse, and Mr. W. Ramsay, all of whom have put in over 40 years' asylum service and to Mrs. Hankinson, head laundress, after nearly 36 years' continuous service. Perhaps if we could learn at what salary and under what conditions of work these gold medallists had served for so many years we might come to understand why the Asylum Workers' Association has failed to convince its clientèle of its benefit to their profession. The last five years have done much to awaken asylum workers to a sense of the remediable hardships of their lot. We believe that when these evils have been remedied, as soon they must be, asylum workers may be ready to respond to a society which has always been conducted with a single eye to raising the tone of the profession, and has done what it could to cheer those who have drawn together under its ægis. We shall look to see the Asylum Workers' Association revive and spread under happier auspices.

The Committee of the Beckett Hospital, Barnsley, have made a further increase in the salaries of the nursing staff. A "substantial increase" has been granted to the matron's salary; that of the assistant matron is now raised to £80. Night sisters start now at £50 and rise to £65; ward sisters start at £45, rising to £60; staff nurses, £35 to £45; probationers, £15, £20, £25. The Beckett Hospital falls into the category of hospitals with an average of fifty to one hundred beds occupied daily, and the salaries suggested by the Salaries Committee of the College for this class of institution are as follows: Matron, £150; assistant matron, £75; sisters, £75, or for wards with only twenty beds, £60; fourth-year charge nurses, £50; probationers, £18, £22, £30. The average salaries actually paid, however, fall considerably below the new Beckett level.

The Committee of the South London Hospital for Women have approved a scheme for a fifty-six hours' working week. When all is in working order at full strength, nurses will have one complete day off in seven. Night-nurses will have four nights off a month, and they will also get one and a-half hours' rest during the night away from the wards. This latter concession is one which, in our judgment, should be made compulsory.

Mrs. Stead, who is relinquishing her connection with the Southwark Day and Night Nursery has offered the institution to the local authority as a going-concern free from all indebtedness, an offer which the Southwark Council's Health Committee proposes to accept. Last year's balance sheet of the nursery shows an income of £720, comprising £280 12s. from the mothers, £393 from the Ministry of Munitions, and £46 9s. from subscriptions. The expenditure was £812, as follows: Wages and insurance, £422 14s. 11d.; food and provisions, £288 17s. 5d.; furniture and equipment,

**ROUND THE HOSPITALS—(*continued*).**

£8 16s. 5d.; printing and postage, 7s. 5d.; fuel, light and cleaning, £78; laundry, £12 11s. 8d.; sundries, 12s. 2d. The local authority cannot expect subscriptions, but it is pointed out that such loss can be made good by charging a fee of 7d. instead of 6d. per child, whilst executive expenses ought easily to be reduced. The Health Committee thinks that the running of the institution would afford an excellent opportunity to obtain valuable experience for the future success of municipal crêches, and would, if successful, be the basis upon which the scheme could be extended over the whole area.

Miss Mary Berwick is leaving the Higginbotham Sick Poor Association, Glasgow (Q.V.J.I.N.), after a service of twenty-six years, during nineteen of which she has been superintendent. The Directors of the Association warmly express their sense of the incalculable value of her services to the sick poor of Glasgow. She has been much loved by the nurses under her, and her influence in the Nurses' Home is appreciated by all. Her well-earned rest after over a quarter of a century spent in the same quiet continuance in good works will be cheered by the good wishes of countless friends. Her successor is Miss S. H. Mitchell, who left her post as Superintendent of the Motherwell Queen's Nurses Association to take up military nursing and has now been released.

The *Graphic* of May 24 gave a delightful illustration of the African Bibi (Lady) Nursing Corps. It was formed in Uganda in 1917, the first to enrol being Fatuma, the wife of a native soldier in the firing line. Other Bibis soon volunteered, and they worked with the utmost goodwill and success. They received a three months' training in nursing and first-aid, and their uniform was a long white coat with short sleeves worn over their native dress, with a white handkerchief covering their woolly plaits. The badge of their corps—A.B.C.—in white on a purple band was worn on the left arm. The Bibis, it is almost needless to say, owed their existence to the initiative of an English nursing sister who took great pride in her pupils. It is hoped they may form the nucleus of a nursing service in the Askari garrison hospitals.

The thoroughness with which every American grave was identified by the American Red Cross, and the fine spirit of gratitude shown in the reverent and loving ceremonies which honoured them on May 30, form a great lesson to the British people. As a nation we are too little mindful of these things. We leave too much to private initiative, and the result is that we discover, often, and indeed very much too often, that there has been neglect. Has it been the business of the British Red Cross Society to see that the graves of nursing sisters who died on war service have been honoured by any mark of recognition? Surely there should be a record of those who lie buried abroad, and care should be taken that once a year at least they should be visited and tended. The Americans leave nothing to chance in fulfilling this pious duty, and we shall do well to learn from them.

Sir Thomas Dewey, presiding at the annual meeting of the Nurses' Insurance Society (the separate section of the Royal National Pension Fund for Nurses), gave an interesting bit of information which illustrates the extent to which the influenza epidemic took toll of the nursing profession last autumn. Up to the end of last September the sickness and disablement benefit had cost the Society £550 less than in the first nine months of the previous year. Yet by the end of December the total paid out in this way to nurses had risen to £1,170 in excess of 1917. Those who are sceptical about the benefits of sick insurance should ponder over these figures.

It is Pound Day at Queen Charlotte's Hospital on June 5, as this issue of The Hospital appears. Princess Arthur of Connaught is attending the Annual Meeting of the Ladies' Association, of which she is Patron, at 3 p.m. on that day, and an urgent appeal has been issued for plentiful gifts to replenish the store-room. It is stated that since war broke out 5,000 sailors' and soldiers' wives have been tended by this hospital.

Founders' Day was kept at the Treloar Hospital for Cripples at Acton on May 31, with ceremony and enthusiasm. Sir William Treloar stated at the luncheon that the proportion of cases successfully treated at the hospital was over ninety per cent., and that they had now a nursery school approved by the Board of Education for children from two to five. Among the most interesting events of the day was the formal opening of the Florence Treloar Ward for Sick Nurses by Sir Edward Clarke.

The Elsie Inglis unit of the Scottish Women's Hospitals sent out to serve the Jugo-Slav Division of the Serbian army on the Macedonian front in February last year has now been recalled, its services being no longer required. Before leaving for home every member of the hospital staff was presented with the Serbian Gold Medal for Meritorious Service.

The 1919 Directory of District Nursing and Streets' List for London has been issued by the Central Council for District Nursing in London. It is one of the advantages of this admirable production that it does not get out of date, for there are few alterations in the district nursing services attached to particular neighbourhoods. In the course of two years it has, however, become apparent that a good many streets have been omitted, and that a few changes have been made in the addresses of the societies. A supplement, as clearly printed and well edited as the rest of the volume, has been added. The Directory is an admirable production, and the small price of 2s 6d. does not nearly cover the cost. It is indispensable to every health visitor, almoner, and philanthropic worker in London.

